# Neutralization of murine myeloid-derived suppressor cells enhances the efficacy of a chimeric antigen receptor T-cells directed against pediatric solid tumors

**DOI:** 10.1186/2051-1426-1-S1-P265

**Published:** 2013-11-07

**Authors:** Steven L Highfill, Adrienne H Long, Rimas J Orentas, Crystal L Mackall

**Affiliations:** 1 Pediatric Oncology Branch, NCI, Bethesda, MD, USA

## 

Genetically engineered T-cells that express chimeric antigen receptors (CARs) to directly target tumor-expressed antigens have shown remarkable activity in clinical trials for hematologic malignancies, but remain unproven for the treatment of solid tumors. We find that in addition to its well known high expression in neuroblastoma, the disialoganglioside GD2 is also highly expressed on pediatric sarcomas. Some human sarcoma cell lines have GD2 expression levels equivalent to that of the prototypical neuroblastoma cell line LAN5, and immunohistochemical staining of primary human tumor tissue samples taken from essentially all patients with metastatic osteosarcoma and a subset of patients with alveolar rhabdomyosarcoma demonstrate robust expression of GD2 on the cell surface. Based on these results, we have developed models to test the ability of GD2-CAR T-cells to target and lyse human sarcomas in vitro and in vivo. We find that GD2-CAR modified T-cells induce specific lysis of GD2-expressing solid tumors in vitro even at effector:target ratios as low as 1:1, but fail to induce a significant response in vivo using the same human cell lines. Interestingly, we discovered that the pediatric sarcoma xenografts induce a large expansion of murine CD11b+Gr1+ myeloid-derived suppressor cells (MDSC) that inhibit human T-cell responses in vitro. These results lead us to adopt a combinatorial therapeutic strategy in which we first neutralized the suppressive potential of MDSC by administration of all-trans retinoic acid (ATRA) followed by GD2-CAR therapy. This combinatorial approach results in significant improvements in both overall survival and tumor growth. Given that retinoids are already available in the clinic, these results suggest that the effectiveness of CAR T-cell therapy for solid tumors could be enhanced by coadministration of retinoids to modulate the myeloid derived suppressor cells.

